# Formulation and Evaluation of Optimized Oxybenzone Microsponge Gel for Topical Delivery

**DOI:** 10.1155/2015/261068

**Published:** 2015-02-18

**Authors:** Atmaram P. Pawar, Aditya P. Gholap, Ashwin B. Kuchekar, C. Bothiraja, Ashwin J. Mali

**Affiliations:** Department of Pharmaceutics, Bharati Vidyapeeth University, Poona College of Pharmacy, Erandwane, Maharashtra 411038, India

## Abstract

*Background*. Oxybenzone, a broad spectrum sunscreen agent widely used in the form of lotion and cream, has been reported to cause skin irritation, dermatitis, and systemic absorption. *Aim*. The objective of the present study was to formulate oxybenzone loaded microsponge gel for enhanced sun protection factor with reduced toxicity. *Material and Method*. Microsponge for topical delivery of oxybenzone was successfully prepared by quasiemulsion solvent diffusion method. The effects of ethyl cellulose and dichloromethane were optimized by the 3^2^ factorial design. The optimized microsponges were dispersed into the hydrogel and further evaluated. *Results*. The microsponges were spherical with pore size in the range of 0.10–0.22 *µ*m. The optimized formulation possesses the particle size and entrapment efficiency of 72 ± 0.77 *µ*m and 96.9 ± 0.52%, respectively. The microsponge gel showed the controlled release and was nonirritant to the rat skin. In creep recovery test it had shown highest recovery indicating elasticity. The controlled release of oxybenzone from microsponge and barrier effect of gel result in prolonged retention of oxybenzone with reduced permeation activity. *Conclusion*. Evaluation study revealed remarkable and enhanced topical retention of oxybenzone for prolonged period of time. It also showed the enhanced sun protection factor compared to the marketed preparation with reduced irritation and toxicity.

## 1. Introduction

For topical application of active agents various novel carrier systems were explored till date in the form of microparticles, nanoparticles, liposomes and cochleates, and so forth. Food and Drug Administration (FDA) has approved wide range of products like Retin-A, Carac cream, MicroPeel Plus, and oil control lotion due to improved efficacy and safety compared to the conventional topical drug delivery systems. Typically, such products form a highly concentrated layer of active ingredients on the skin which results in skin irritation and toxicity [1–4].

At present, the need of an exclusive delivery system has been required for the topically active sunscreen agents. Microsponge is sponge-like porous polymeric system in the range of 10–25 *μ*m with extended release of drug. It offers increased payload of drug and stability with reduced irritation, mutagenicity, allergenicity, and side effects for the topical applications. Due to these undue advantages, in the current era of topical products, microsponge has got wide acceptance. Microsponge for topical route has been reported for the controlled release of benzyl peroxide, mupirocin, retinoid, and 5-fluorouracil which reported minimal penetration through dermis, less drug accumulation, and irritation without sacrificing the efficacy of drug [5–7].

The ultraviolet (UV) rays responsible for sunburns increase the risk of basal cell carcinoma and malignant melanoma. These harmful UV radiations may be blocked by either absorption, reflection, or scattering of rays. The various actives were reported to avoid the exposure to harmful UV radiations. Based on the protective action, UV rays blocking agents are broadly divided into physical agents like zinc oxide and titanium dioxide and chemical agents like benzyl cinnamate and cinnamate derivatives, p-aminobenzoic acid, butyl methoxy dibenzoyl methane, and related compounds [8].

Oxybenzone is broad spectrum sunscreen agent widely used in the form of lotion and cream. It acts by absorbing entire UV-B radiations (280–320 nm) and mimics the energy conservation of law. The present oxybenzone loaded sunscreen formulations have been reported to cause skin irritation, photo allergic contact dermatitis, and systemic absorption [9, 10]. Therefore, it was hypothesized that controlled release of oxybenzone from microsponge offers a sophisticated way to increase the light absorbance capacity with reduced side effects and toxicity. This may be responsible for increasing the sun protection factor (SPF) of the formulation.

This is the first attempt to investigate the feasibility of oxybenzone loaded microsponge gel for enhanced sunscreening efficiency with reduced skin irritation and toxicity. The objective of the present study was to formulate oxybenzone loaded ethyl cellulose (EC) microsponge by quasiemulsion solvent diffusion method by 3^2^ factorial design. The optimized formulation was evaluated for physiochemical characterization, percentage yield, particle size, percent entrapment efficiency (%EE), drug release, surface topography, and differential scanning calorimetry (DSC). The optimized microsponge was incorporated into hydroxy propyl methyl cellulose (HPMC-K4M) gel. Further, prepared gel was evaluated for rheological characterization, skin irritation, minimal erythemal dose (MED), and sun protection factor (SPF) determination.

## 2. Materials and Methods 

Oxybenzone was provided by Nulife Pharmaceuticals Ltd., Pune, as a gift sample. Ethyl cellulose-N10, hydroxy propyl methyl cellulose, was gifted by Emcure Pharmaceuticals Pvt. Ltd. Polyvinyl alcohol, dichloromethane, and methanol were purchased from Emerck (India) Ltd., Mumbai.

### 2.1. Preparation of Oxybenzone Loaded Microsponge

All the microsponge formulations were prepared using quasiemulsion solvent diffusion method. Required amount of oxybenzone (0.1% w/v) and EC (0.1–0.3% w/v) were dissolved in dichloromethane (DCM) (3–5 mL). The DCM solution was gradually added in 25 mL of aqueous solution of poly vinyl alcohol (PVA) (0.1% w/v) at room temperature with continuous magnetic stirring. Then the final mixture was filtered to separate formed microsponges and dried in the vacuum drier for 24 h.

### 2.2. Optimization of Formulation

A prior knowledge and understanding of the process variables under investigation led to preliminary experiments. Based on this preliminary data (not shown), the 3^2^ factorial design was adopted to optimize the amount of EC and DCM as the independent variables. The particle size, percent drug entrapped, and the drug release were considered as dependent variables. The response surface graphs of the obtained results were also plotted. The coded and actual values are given in Table 1. The obtained data was analyzed by the results observed from the multiple regression analysis using Design Expert 8.0.6.1 software (Stat-Ease Inc., USA).

The following equation was obtained:

(1) Y=βo+β1X1+β2X2+β11X1X1+β22X2X2+β12X1X2,

where *Y* is the measured response, *X* is the levels of independent factors, and *β* is the regression coefficient.

*X*_1_ and *X*_2_ indicate the amount of EC and DCM.

The optimized microsponge formulation was incorporated in 3% w/w HPMC base to obtain the gels.

### 2.3. Characterization of Optimized Oxybenzone Loaded Microsponge

#### 2.3.1. Determination of Production Yield

Percentage yield is determined by calculating the initial weight of raw materials and the weight of microsponge. Percentage yield was calculated by using the following formula:

(2) $$\begin{array}{c} \text{Percentage}\;\text{yield}=\frac{\text{Practical}\;\text{yield}}{\text{Theoretical}\;\text{yield}}\times 100. \end{array}$$

#### 2.3.2. Microsponge Size Measurement

The mean particle size of the microsponge dispersion was determined by polarising microscope (model number E600POL). The scale was used which was numbered from one to ten microns, each unit corresponding to one micron.

#### 2.3.3. Percentage Entrapment Efficiency Analysis

For the analysis of entrapped oxybenzone, microsponge was dispersed in methanol to release the entrapped drug. The unentrapped oxybenzone was separated from the microsponge by centrifugation. For this, the dispersion was transferred into Eppendorf tube and centrifuged at 20,000 rpm for 1 hr at 4°C. The free drug remained in supernatant while entrapped drug was retained in the pellet. The supernatant, with subsequent dilution and filtration, was then analyzed for drug concentration spectrophotometrically at 287 nm. The percent entrapment efficiency (%EE) was calculated as the following equation:

(3) %EE=Amount  of  drug  entrapped  in  the  microspongeTotal  amount  of  drug  used×100.

#### 2.3.4. Drug Release Studies

To know the rate and extent of drug release from microsponge, dissolution of oxybenzone loaded microsponge was studied using USP dissolution test apparatus (USP XXIII). Accurately weighed samples of microsponge equivalent to 25 mg of oxybenzone were placed in 900 mL of phosphate buffer pH 7.4 and were subjected for dissolution with a paddle speed of 150 rpm at 37 ± 0.5°C. Aliquots (5 mL) were withdrawn at 5 min initially and then at hourly intervals up to 8 hours and assayed spectrophotometrically at 287 nm. The percentage of drug released at various time intervals was calculated and plotted against time [11, 12].

#### 2.3.5. Scanning Electron Microscopy (SEM)

Surface topography of the selected optimized formulation was characterised using scanning electron microscopy (SEM). Freshly prepared microsponge samples were mounted on the aluminium stub and coated with gold-palladium layer by autofine coater (Joel, JFC, Tokyo, Japan) and analyzed with a scanning electron microscope (Joel, JSM-6360, Tokyo, Japan) operated at a 10 kV acceleration.

#### 2.3.6. Differential Scanning Calorimetry (DSC)

DSC studies were carried out using Mettler-Toledo DSC 821e instrument equipped with an intercooler (Mettler-Toledo, Switzerland). Indium and zinc standards were used to calibrate the DSC temperature and enthalpy scale. The samples were hermetically sealed in aluminium crucibles and heated at a constant rate of 10°C/min over a temperature range of 25–300°C. Inert atmosphere was maintained by purging nitrogen gas at flow rate of 50 mL/min.

#### 2.3.7. Rheological Characterization

Rheological measurements of the microsponge loaded gel and blank gel were performed using a controlled stress rheometer (Viscotech Rheometer, Rheological Instruments AB, Lund, Sweden). Data analysis was done with stress rheological basic software, version 5.0. A cone and plate geometry was used with 25 mm diameter and cone of 1.0° [13]. Fresh sample was used for test and all measurements were carried out in triplicate at 25°C and at 37°C.

#### 2.3.8. Creep Recovery Test

In creep recovery, samples were subjected to a fixed stress from LVR for 100 s and then allowed to recover. The creep compliance, *J*, was recorded against time.

#### 2.3.9. Skin Irritation Test

Skin irritation test of optimized oxybenzone loaded microsponge gel (M9) was compared with the marketed and placebo gel. The present study was employed in the three groups of rats (*n* = 6) to evaluate skin irritation. They were kept carefully following an acclimation period of 7 days to ensure their suitability for the study. Test animals were kept within a limited-access rodent facility with environmental conditions set to a temperature of 25 ± 2°C, a humidity of 60–90% RH, and a 12-h light/12-h dark cycle. Animals were provided* ad labium *access to a commercial rat diet and drinking water was supplied to each cage. The area on the back of each rat was shaved prior to the experiment. The first group of the rat was applied with microsponge gel and the second group was applied with commercial sunscreen lotion. The remaining group of rats was considered as a control group.

The 0.5 g of each test product was placed on each area (25 × 25 mm) for 30 minutes. Finally treated skin area of rats was washed off by tap water. Scoring of the erythema was performed at 24 and 72 hours and both the treated and controlled sites were covered and wrapped by cotton bandage. Reactions on skin were measured after 24 hr and 72 hr in form of erythema. The mean erythemal scores were recorded (ranging from 0 to 4) according to Draize scale [14]. The responses of all the formulations applied on rat skin surface were evaluated and primary irritation index (PII) was calculated and matched with response category as shown in Table 3. The score of primary irritation was calculated for each rat. Scores for erythema at 24 and 72 hours were summed and divided by the number of the observations for the treated sites [15].

#### 2.3.10. Determination of Minimal Erythemal Dose (MED)

The study was performed using Wistar rat model. This model is suitable for the study because of the photochemical changes taking place in rat skin after UV exposure. Before conducting actual SPF testing, a study was carried out to determine MED with respect to time of unprotected skin and protected skin of Wistar rat. This was carried out on total of nine rats. They were divided into three different groups each comprising three rats and maintained separately. A solar simulator high pressure mercury vapour lamp (Osram Ultra Vitalex of 300 w) was used as a UV light source. First group of rats was kept directly under the solar simulator lamp and was sampled after every one minute. The rats in the second and third group were applied with marketed sunscreen lotion and oxybenzone loaded microsponge gel, respectively, and the same sampling procedure was followed. The presence of reddening (erythema) of skin was noted after 3 hr of completion of study.

#### 2.3.11. SPF Testing

The* in vivo *analysis was performed to study the effectiveness of the optimized formulation (M9) in comparison to the marketed formulation. All studies were approved by the institutional animal ethics committee of Poona College of Pharmacy (1703/PO/c/13/CPCSEA), Pune, India, and were conducted under the provisions of the approved protocol. According to the personal care product council (PCPC), formerly Cosmetics, Toiletries and Fragrance Association (CTFA) and European Cosmetic Toiletry and Perfumery Association (COLIPA), SPF determination protocol, the amount of sunscreen formulation to be applied should be 2 mg/cm^2^ [16] and the same was used in present study. The SPF was determined by using the following formula:

(4) $$\begin{array}{c} \text{SPF}=\frac{\text{MED}\left(\text{PS}\right)}{\text{MED}\left(\text{US}\right)}.\;\; \end{array}$$

MED (PS) = minimal erythemal dose of protected skin; MED (US) = minimal erythemal dose of unprotected skin.

## 3. Results and Discussion 

### 3.1. Preparation of Oxybenzone Loaded Microsponge

During the preliminary study, concentration of EC and DCM which would produce nonaggregating, nonregimenting, and porous microsponge dispersion was determined. The predicted concentration of EC and DCM was decisive in the preparation and stabilization of microsponge. EC was added to provide structural integrity and PVA was added as an emulsifying agent. The concentration of DCM showed influence on EE and porosity. The preliminary data suggested that the concentration of PVA required was 100 mg. This was kept constant. Altering the concentration of EC and DCM caused pronounced effect on microsponge dispersion.

### 3.2. Optimization of Independent Variables of Microsponge Formulation

The concentration of PVA and oxybenzone was kept constant. The effects of EC and DCM on the microsponge size, entrapment efficiency, and* in vitro *release were analyzed.

### 3.3. Characterization of Oxybenzone Loaded Microsponge

#### 3.3.1. Effect of Variables on Microsponge Size

The size of the microsponge ranges from 72 ± 0.77 to 619 ± 0.02 *μ*m which has been shown in Table 1. The most important parameter which needs to be monitored during microsponge preparation for its best performance was the size of microsponge. Thus for the effective topical application, the selected method should result in optimum size range and homogeneous microsponges. In the present study, quasiemulsion solvent diffusion method was found to produce uniform microsponge. The obtained microsponge has narrow size of distribution. It was observed that the relative amount of EC and DCM was found to play an important role in determining size of microsponge which was found to be in the range of 100–600 *μ*m. The multiple regression analysis for the mean particle size of factorial batches revealed the good fit (*R* ^2^ = 0.8429) as shown in (5). The effect of both independent variables on the size of the particle was given by the following equation:

(5) $$\begin{array}{c} Y1=276-138X_{1}-127X_{2}+67X_{1}X_{1}-76X_{2}X_{2}+30X_{1}X_{2},\\ R^{2}=0.84. \end{array}$$

**Figure 1:**
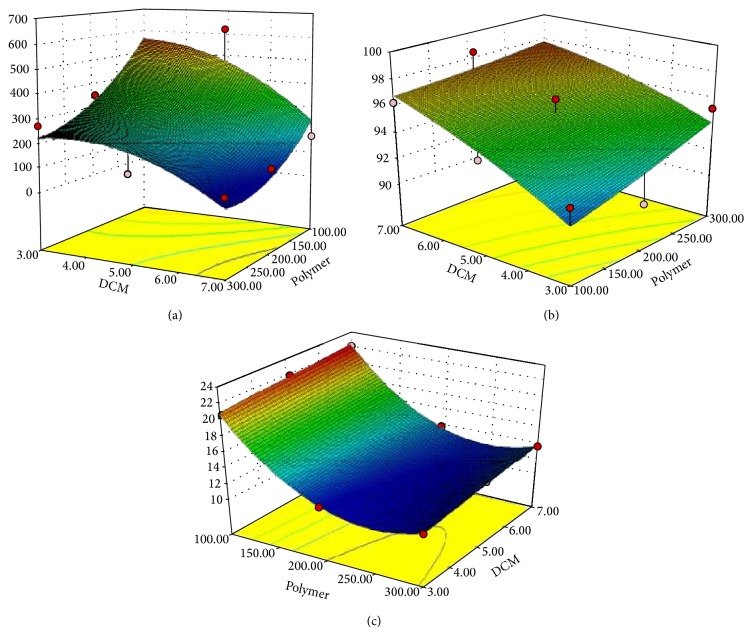
Response surface plots of (a) particle size, (b) entrapment efficiency, and (c) drug release.

According to (5) and Figure 1(a), *X*_1_ indicates negative effect on particle size which may be due to increase in concentration of EC that leads to an increase in the intramolecular cohesive forces. These forces may be reduced due to the surfactant property of the PVA which further leads to increase in the microsponge size [16]. However, the interaction term *X*_1_ *X*_1_ is positive supporting the fact that increase in polymer concentration leads to increase in the viscosity of the internal phase; thus bigger globules are formed during emulsification leading to an increase in mean particle sizes.

The negative value of *X*_2_ indicates that an increase in DCM concentration reduces the particle size. When the dispersed phase with lower viscosity (batch M9 contains 7 mL DCM) was poured into the continuous phase, the globules of the formed emulsion could be subdivided into smaller particles [1, 17, 18].

The interaction term *X*_2_ *X*_2_ is negative supporting the fact that increase in DCM concentration leads to decrease in the viscosity of the internal phase; thus smaller globules are formed of the emulsion leading to a decrease in mean particle sizes. The interaction terms *X*_1_ *X*_2_, that is, combined effect of concentration of EC and DCM, have a positive influence on particle size. This may be because of increase in the viscosity of internal phase with polymer concentration where the globules of formed emulsion can hardly be subdivided into smaller particles [19, 20].

#### 3.3.2. Effect of Variables on Entrapment Efficiency

It is general observation that increase in the polymer ratio increases the EE. The reason for increase in EE with high polymer ratios is reduced diffusion rate of drug solution from concentrated polymeric solutions into external phase. This provides more time for the droplet formation and may improve the yield of microsponge and entrapment efficiency. The equation was generated by fitting the observed coefficient in (1) as follows:

(6) Y2=95.29+1.03X1+2.33X2+0.12X1X1−0.47X2X2−0.55X1X2,R2=0.75.

According to results of EE as shown in (6) and Figure 1(b), the higher drug concentration was obtained at the higher polymer ratios which was above 95% as shown in Table 1. When DCM diffuses out, nearly all of the dispersed phase is converted to solid microsponge particles.

The positive value of *X*_1_ indicates highest drug EE in the microsponge. This can be explained by the fact that the amount of drug per unit of polymer was greater than other microsponge formulations. The reduced diffusion rate of DCM from the concentrated solution results in more time for droplet formation with increased precipitation of the drug in the microsponge. This leads to increased %EE [2, 21]. It was also observed by positive value for the interaction terms *X*_1_ *X*_1_. The value of *X*_2_ has a positive influence on the %EE. This may be due to the higher concentration of DCM which provides more solvent action. Moreover the polymer-drug association was also delayed molecular evaporation of the solvent which leads to increased drug precipitation in the microsponge [1]. However the interaction term *X*_2_ *X*_2_ has a negative influence on the %EE. This may be due to the leaching of the drug into the aqueous phase from more spongy and porous microsponges.

The interaction term *X*_1_ *X*_2_ has a negative influence on the EE. This may be observed due to increased intramolecular cohesive forces which cause the densification of core part of the microsponge. This leads to leaching of drug towards periphery of microsponge and in the external phase [14, 22].

#### 3.3.3. Effect of Variable on Drug Release

The second most important parameter which needs to be monitored during microsponge preparation for its best performance was the release of the drug from the microsponge. The oxybenzone should be released slowly over the extended period of time to modulate low concentration gradient for skin transport. Therefore, multiple regression analysis for the drug release as per the factorial design revealed the good fit *R* ^2^ = 0.99 with the following equation:

(7) Y3=12.96−4.33X1+0.76X2+4.36X1X1−0.04X2X2+0.013X1X2,R2=0.99.

As per (7) and Figure 1(c), the correlation coefficient indicates a good fit. The negative value of *X*_1_ indicates decrease in the drug release because of increase in the concentration of EC [17]. The higher amount of EC leads to formation of thick layers which may be responsible for the controlled release of drug [22]. The variable *X*_2_ has a positive influence on the drug release. This is attributed to the fact that higher concentration of DCM yields more spongy and porous microsponge. The increased amount of DCM also causes the precipitation of the drug at the periphery of the microsponge. This leads to increase in drug release as shown in (7) [2].

**Figure 2:**
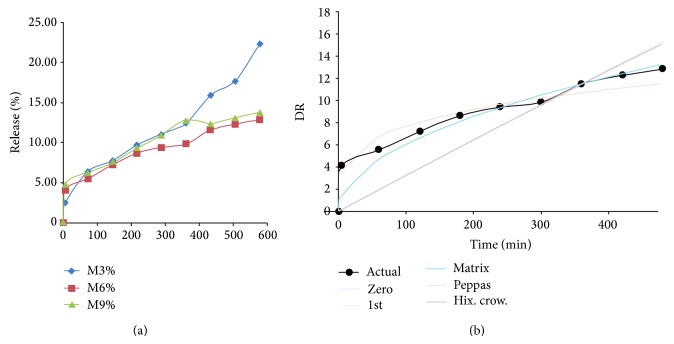
(a) Comparative* in vitro* release profile for M3, M6, and M9 formulations and (b) release kinetics for M9 formulation.

The positive value of interaction term *X*_1_ *X*_1_ can be supported by increase in the intramolecular cohesive forces as explained previously. The migration of drug towards periphery of microsponge may cause faster drug release. The interaction terms *X*_2_ *X*_2_ and *X*_1_ *X*_2_ have a negligible effect on drug release. The effect of polymer ratio on the release of the drug from microsponge containing 100 mg, 200 mg, and 300 mg microsponge was shown in Figure 2(a), respectively. Different kinetic models were employed to fit the data relating to the kinetics of oxybenzone from microsponge. The results shown in Table 2 depicted the release kinetics on the basis of the *R* ^2^ values. The results indicated best fit of Higuchi model for M6 and M9 formulation. The is because M3 has lower ratio of polymer that makes the walls of the microsponge thinner compared to that in M6 and M9 and release takes place by erosion diffusion at a faster rate. M9 was selected for further deposition studies as it has the diffusion release pattern, maximum %EE, and smallest and uniform particle size of 72 *μ*m [22–24].

#### 3.3.4. Scanning Electron Microscopy

**Figure 3:**
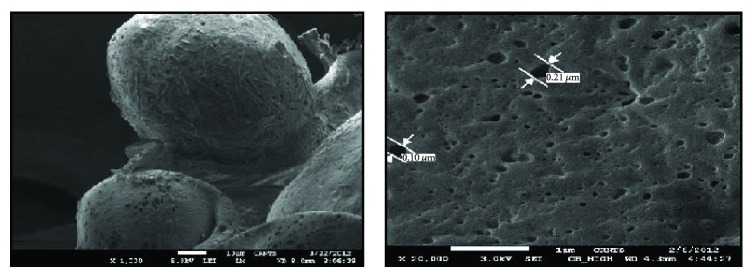
Microsponge with surface pore size.

The SEM of microsponge was shown in Figure 3. As shown in the SEM, it revealed spherical microsponge with surface pores. The observed pore size was within the range 0.10–0.22 *μ*m.

#### 3.3.5. Differential Scanning Calorimetry

**Figure 4:**
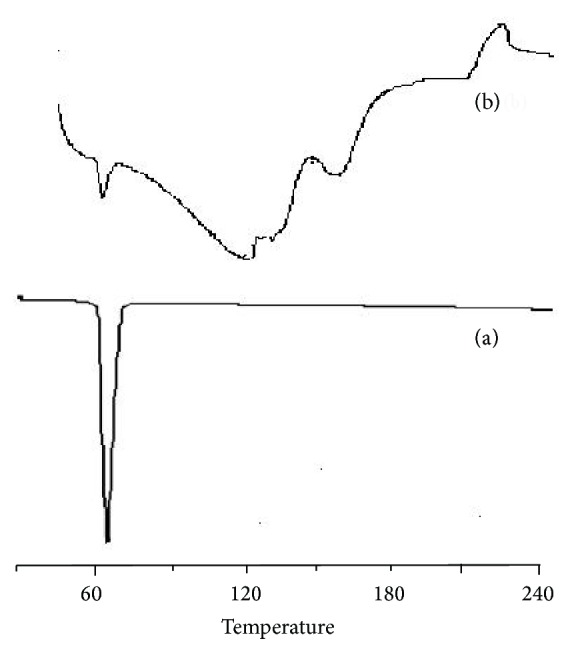
DSC plots of (a) pure drug and (b) formulation.

As shown in Figure 4, oxybenzone dispersed in EC showed the same thermal behaviour as drug. The exothermic peak was observed at 64°C which corresponds to the melting point of the pure drug. This report indicates that the thermal properties of oxybenzone were not altered during formulation of microsponge.

#### 3.3.6. Preparation of Microsponge Loaded Gel

The optimized microsponge formulation (M9) was incorporated in 3% w/w HPMC base to obtain the gels. Initially required amount of HPMC was added to water and kept overnight for complete hydration of polymer chains. Microsponge dispersion was added to the hydrated HPMC solution to obtain a final concentration of 2.5% w/w of oxybenzone. The prepared gel was used for drug deposition study.

#### 3.3.7. Rheological Characterization

**Figure 5:**
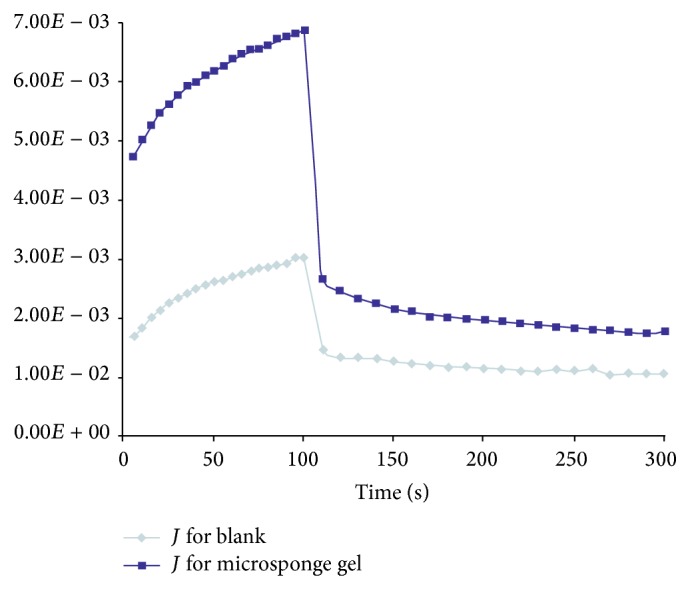
Creep recovery of blank and drug loaded microsponge gel.

The microsponge gel showed creep recovery of 74.05% compared to 65.11% of blank gel. The same results were depicted in Figure 5. However from the observed results, oxybenzone loaded microsponge gel showed higher elastic contribution than viscous one. In the creep recovery test, it has shown highest creep recovery which was helpful for promising gelling with higher elasticity. This property helps in thinning of the gel under pressure with higher adherence time on the skin.

#### 3.3.8. Skin Irritation Test

Skin irritation test of optimized oxybenzone loaded microsponge gel (M9) was compared with the marketed and placebo gel. Erythema with score of 0–2 was observed on all rats. After 72 hours, disappearance of all erythema was observed from test areas applied with placebo gel and microsponge gel as shown in Table 3. The values for PII for placebo gel and microsponge gel were in the range of 0.1–0.4 indicating negligible irritancy. The microsponge gel has shown 0.375 of PII. The PII for marketed lotion was found to be 1.12 indicating slight irritation. From the obtained results it was clear that the microsponge gel for topical delivery has achieved the objectives of controlled drug release. The microsponge is too large to pass through the stratum corneum and hence it would be expected to remain on the skin surface, gradually releasing its contents over time. This reduces higher exposure of oxybenzone to the skin and contact period. This was helpful to reduce the irritation and toxicity of drug.

#### 3.3.9. Determination of Minimal Erythemal Dose

The time required for the development of erythema for the unprotected rats was 7 min. The rats protected by the marketed formulations showed 140 mins for the development of erythema. The microsponge gel showed the 170 min which was more as compared to marketed formulation. This result was maybe observed due to the controlled release of drug from the microsponge loaded gel which helped to avoid excessive deposition of drug.

#### 3.3.10. SPF Testing

**Figure 6:**
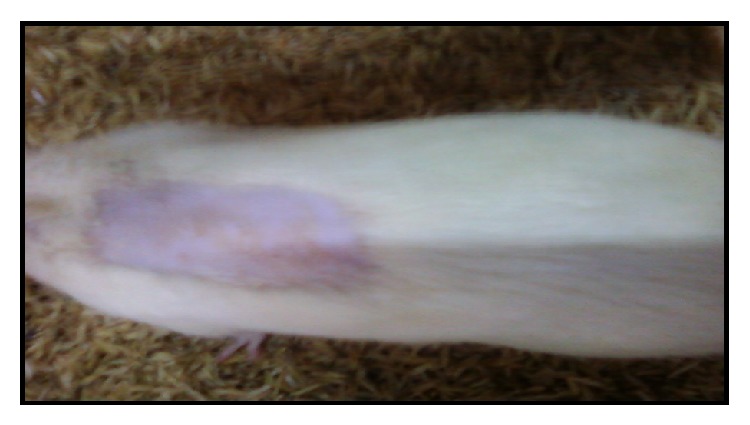
Protected skin applied with M9 microsponge gel.

The SPF determination of optimized oxybenzone loaded microsponge gel (M9) was compared with the marketed gel. The microsponge gel showed SPF 25 as compared to SPF 20 of the marketed lotion. This was maybe due to slower release of drug from the microsponge gel which provides prolonged retention of oxybenzone. This was reflected in the comparison with marketed lotion where the oxybenzone loaded microsponge gel was found to be more photoprotective. Thus, as shown in Figure 6, topical application of microsponge gel provides more efficacy and safety by overcoming skin irritation and toxicity problem.

## 4. Conclusion

The present study concluded successful preparation and optimization of oxybenzone loaded microsponge gel with the enhanced sunscreening efficiency.* In vitro *and* ex vivo *evaluation study of microsponge gel revealed remarkable and enhanced topical retention of drug for prolonged period of time with increased SPF compared to the marketed preparation. A controlled release of oxybenzone onto the skin over a prolonged period of time was beneficial for enhanced UV protection efficacy. This study provides future insights for developing controlled release microsponge gels for topical applications containing sunscreen agents.

## Figures and Tables

**Table 1:** Factorial design and characterization of experimental formulations.

Formulations (EC, DCM) (mg, mL)	% EE ± SD	% DC ± SD	% production yield	Particle size *d* (0.9) *µ*m ± SD	% DR ± SD
M1 (100, 3)	92.3 ± 0.12	67.12 ± 0.42	50.14 ± 0.28	496 ± 0.12	20.08 ± 0.02
M2 (100, 5)	93.7 ± 0.22	70.00 ± 0.56	59.76 ± 0.10	619 ± 0.02	21.08 ± 0.82
M3 (100, 7)	98.6 ± 0.14	73.54 ± 0.82	73.54 ± 0.16	146 ± 0.32	22.28 ± 0.72
M4 (200, 3)	90.1 ± 0.21	44.26 ± 0.42	44.10 ± 0.14	347 ± 0.42	12.19 ± 0.42
M5 (200, 5)	96.3 ± 0.56	67.54 ± 0.32	67.76 ± 0.72	224 ± 0.52	12.88 ± 0.32
M6 (200, 7)	98.5 ± 0.72	54.28 ± 0.22	55.22 ± 0.32	105 ± 0.62	13.74 ± 0.42
M7 (300, 3)	95.3 ± 0.92	65.33 ± 0.29	65.11 ± 0.42	272 ± 0.02	12.23 ± 0.02
M8 (300, 5)	96.7 ± 0.62	42.02 ± 0.12	42.78 ± 0.62	119 ± 0.28	12.94 ± 0.11
M9 (300, 7)	96.9 ± 0.52	77.87 ± 0.92	77.56 ± 0.20	072 ± 0.77	13.76 ± 0.10

**Table 2:** *In vitro* release kinetics of optimized formulations.

Formulation	Kinetic model	*R* ^2^ value
M3	Peppas equation	0.97 ± 0.09
M6	Higuchi equation	0.96 ± 0.12
M9	Higuchi equation	0.95 ± 0.11

**Table 3:** Skin irritation test.

Animal number	Reaction	Placebo gel (24 h)	Placebo gel (72 h)	M9 gel (24 h)	M9 gel (72 h)	Marketed lotion (24 h)	Marketed lotion (72 h)
1	Erythema	0	0	0	0	1	1
2	Erythema	0	0	1	0	2	1
3	Erythema	1	0	1	0	1	1
4	Erythema	0	0	1	0	1	1
Primary irritation index (PII )	—	1/8 = 0.125		3/8 = 0.375		9/8 = 1.12	
	—	Negligible irritation		Negligible irritation		Slight irritation	
